# Conservative Treatment of Inflammatory Diseases of the Uterine Appendages and Sequelæ by Electricity

**Published:** 1890-03

**Authors:** Augustin H. Goelet

**Affiliations:** New York; Philadelphia, Pa.


### THE CONSERVATIVE TREATMENT OF INFLAMMATORY DISEASES OF THE UTERINE APPENDAGES AND SEQUELÆ BY ELECTRICITY.*

BY AUGUSTIN H. GOELET, M. D., PHILADELPHIA, PA.

MR. PRESIDENT AND GENTLEMEN—Thanking you for the honor conferred upon me by this invitation, I am at the same time much pleased to be able to talk with you upon a subject in which I am deeply interested.

There is no condition in the whole range of gynecological disorders which requires more thoughtful and careful consideration than inflammation of the uterine appendages and its sequelæ. To the patient who suffers it is certainly a subject of paramount importance, and to the physician it is a question of great moment as to what course he shall advise. Shall he try to cure the disease, or shall he take the shorter and more popular course, and advise removal of the diseased organs, thereby confessing his inability to effect a cure? By the older methods of treatment at his disposal, it is true that some cases have been cured; but more frequently the condition has remained unchanged in spite

*Read before Philadelphia Obstetrical Society, January 2, 1890.

of treatment, the pain persisting so obstinately that the patient, as well as the physician, is driven ultimately to a laparotomy for the hope of relief. But alas! how often have both been disappointed in their expectations, the same old pain, or something worse, being her only recompense for the risk which she has undergone, to say nothing of the permanent sterility which is induced if the operation includes both ovaries. It is true that the mortality following this operation has been so much reduced as to make it appear encouraging; but who will gainsay that there is risk, even in the hands of the most experienced operators? And who will affirm that the results obtained, in those who recover from the operation, are entirely satisfactory in more than half the number operated upon? Viewed from this standpoint, is the picture calculated to encourage the conscientious gynecologist?

When we are offered a remedy which promises to cure many of these conditions which were formerly thought incurable, it is obviously our duty to give it a trial before we condemn poor suffering women to a fate which we may never cease to regret. It is nothing new, and for this reason many are skeptical; but the principles upon which the method is based are sound and scientific, and the statistics of results are generally good. But it is a method which must be understood to be appreciated. I look upon those who cannot accept it with much sympathy, for I once stood in the same ranks, and was very unwilling to believe. But, gentlemen, "seeing is believing." Electricity has wrought some wonderful cures. I have seen accomplished with this agent what ten years ago would have been thought the dream of a lunatic. Obstacles which were then insurmountable are now matters of every-day accomplishment. For example, I do not believe I ever cured a bad chronic endometritis until I learned how to do so with electricity; and I believe there are many of you present who will acknowledge that they have discouraged themselves as well as their patients with the application of iodine, glacial acetic acid, nitric acid, etc. And how many of these cases really got worse instead of better under that form of treatment?

There are cases being cured every day by electricity which would otherwise be submitted to the knife, and the time is in the near future when it will be the acknowledged duty of the abdominal surgeon, either to give or cause to be given an extended trial of electricity in every case before laparotomy will be considered justifiable, except in those cases, fortunately rare, which call for immediate operation, where delay means certain death. But to-day how different is the custom! It frequently happens that laparotomy is the first thing suggested. Who will say that this operation is not abused?

Do not misunderstand me. I do not discourage the operation when it is necessary, but wish to urge more conservative methods than exist at the present day.

But do not be misled by thinking that the advocates of electricity claim it will do everything, or that no case can resist its charmed influence. This is a perversion of the truth which its enemies proclaim when they meet a case that has not been benefited by it. Candidly, we acknowledge that there are cases which cannot be benefited even by electricity, and until medical science has become more perfect, they must submit to the inevitable.

In describing the treatment of inflammation of the uterine appendages, I will take up three stages separately.

*Acute Stage.*—Apostoli extols the virtue of vaginal bipolar faradization from the long, fine wire or current of tension in this stage; but I have had no practical experience with it here, and can therefore express no opinion of its merits. Judging, however, from the marked sedative effect which I have obtained from it in the subacute stage, I would have no hesitancy in employing it in the acute stage should an opportunity occur. My experience with this method of using the faradic current dates only from my recent visit to Paris; but I was impressed with what I saw of its effects while there, and my experience with it since has justified the good opinion formed of it.

Let me say here, that it is impossible to get this sedative effect from the faradic batteries of ordinary construction. It is necessary that the current should be a purely induced one, and that the wire on the bobbin should be very fine and have great length.

This bobbin should slide easily over the primary coil around the bundle of soft wires. The current should start at nothing, with the bobbin at a distance from the primary coil, and should be so gradually increased by sliding the bobbin over the primary coil that the patient scarcely feels the increase, and at no time should she experience the least pain from the current. The *seance* should last until perfect sedation is accomplished and she expresses herself free from pain, and the applications should be repeated often enough to keep the pain completely under control; so that, when necessary, they must be repeated several times a day.

*Subacute Stage.*—In this stage I am prepared to speak of bipolar faradization from clinical experience in the highest terms of praise. The harassing pain which yields so unsatisfactorily to the ordinary methods of treatment can be promptly relieved by this method, if carefully and systematically carried out. The applications should at first be vaginal, and should be used daily for from fifteen to twenty-five minutes at a time until perfect sedation is accomplished. The abdomen, which in some of these patients can barely be touched previous to the application, will allow any amount of handling afterwards. The pain is relieved for only a few hours perhaps after the first application, but with every succeeding *seance*, it will be relieved for a longer period, until eventually the relief will be prolonged until the next application, twenty-four hours later. In some instances, the first application will afford perfect relief for several days at a time. With the cessation of the pain, the associated congestion is likewise relieved; for this current, used in this way, has a decided local effect upon the circulation in equalizing it. The same precautions necessary in the acute stage are to be observed in this; the current must be increased very gradually, and it should never be used strong enough to produce pain, and should be continued until perfect sedation is accomplished.

When all that is possible has been accomplished by these vaginal applications, and when the uterus will tolerate the introduction of the sound, the small bipolar uterine electrode, curved to suit the canal, is introduced into the uterus, and the applications are made there in the same way.

When this stage begins to merge into the chronic state, the treatment must be reënforced by intra-uterine galvanization with the positive pole. At first only an occasional application is given, say once or twice a week, of not more than from 20 to 30 M., for three or four minutes, and the faradization is continued in the interval.

If the patient's confidence can be gained at the outset by relieving her pain, half the battle is already won, and this can certainly be done by fine-wire bipolar faradization.

*Chronic Stage and Sequelæ.*—In the chronic stage, the constant or galvanic current is used, and to accomplish results it is necessary to employ apparently heroic doses. But it must be understood that tolerance of the current is progressive, and that by gradually increasing the strength it is possible, eventually, to use with safety a dose which at first would not only be unsafe but unbearable to the patient. This is a point which cannot be clearly understood except by those accustomed to the use of this agent. The large size of the external electrode allow these apparently enormous doses to penetrate the skin and to be borne by the patient with little or no inconvenience.

In some conditions it is possible to accomplish much with the vaginal applications, if used sufficiently strong; but the small doses here are ineffectual, and mere waste of time.

I will speak more particularly of the use of the intra-uterine chemical galvano-caustic applications and of the vaginal galvano-puncture, the only way this current is used by Apostoli in these conditions.

The *positive* pole is always the choice in commencing this treatment when any degree of inflammation exists, because it is less irritating and exciting than the other pole, and exerts a more sedative influence. Beginning with not more than 20 to 40 M., with the bare platinum electrode, which is so arranged as to take effect upon the whole uterine canal, from the eternal *os* to the fundus, the first *seances* should last from three to five minutes, and be repeated every second day. Gradually increasing the dose at the succeeding *seances* as tolerance is established, the current is pushed when necessary and well borne to 50, 100, or 150 M. But when the higher intensities are reached it is better to repeat the applications only once or twice a week. Too frequent applications sometimes produce a sensitive condition of the endometrium, which prohibits further applications for a time, until this has subsided. There is always associated with inflammatory conditions of the appendages a sensitive condition of the endometrium, which will sometimes restrict the increase of the dose beyond 50 or 60 M. And in some cases it is quite unnecessary to exceed this dose, for it will often accomplish all that is desired.

When the case does not progress satisfactorily, nor bear the intra-uterine treatment well, or when all good possible has been accomplished by the intra-uterine applications, the *positive* vaginal galvano-puncture is done. A small fine-pointed needle is used, which is not insulated as it passes through the tissues, and the puncture is made to a depth of only half a centimetre—just enough to enable the current to gain access to the disease. Marked benefit will follow these punctures if the indication is appropriate; and it is seldom necessary to exceed 50 M. used for five minutes, which often the patient can bear without an anæsthetic. When necessary, a very little chloroform may be given to take off the rough edge, for punctures are more painful than the intra-uterine applications. Usually the benefit from a puncture of this kind will be at once noticeable. A sensitive spot in the pelvis, which previously was so tender that the slightest touch caused the patient to flinch, will afterwards be so changed as to allow considerable manipulation. The patient may suffer an aching in the pelvis for a varying period during the first twenty-four hours, but after this passes off, she begins to notice a decided improvement in her previous condition, which continues. In some cases, one puncture will be sufficient, the cure being completed by the intra-uterine applications. A wonderful change is often brought about,—the previous sensitive condition has disappeared, and with it the fulness or thickening in the broad ligament, which was plainly noticeable to the touch before. One puncture will not always be sufficient, and when others are necessary they may be done at intervals of a week.

The method of procedure is this, viz.: The vagina and vulva are douched with an antiseptic solution; the insulating sheath, which covers the needle, is passed along the finger to a point previously selected. Care must be observed to avoid a pulsating vessel, and the lateral surface of the vaginal vault is to be preferred. Selecting the most sensitive spot then, with these precautions, the sheath is held firmly against it, and the needle is passed through it and into the tissues to the limit previously fixed by a set screw on the handle of the instrument. Apostoli uses a steel needle, and destroys one every time by the action of the positive pole. I have had a needle made with a steel shank and a platinum point which screws into it. This needle will last for some time, when the point will become dulled and require sharpening. After the application, and when the needle has been withdrawn, a loose iodoform or creolin gauze tampon is placed in the vagina, and renewed every twenty-four hours.

The positive puncture is to be preferred where it is effectual, because it provokes less irritation. Under its influence, absorption is promoted, though in a less degree than by the negative pole. Even in exudations, especially if recent, the positive puncture will promote and hasten absorption, but some exudates require the negative puncture, which is more powerful to effect absorption.

While the intra-uterine galvanization has for its object the accomplishment of a cure of the inflamed appendages by attacking first the inflammation of the endometrium, which almost always co-exists and which in many cases has been the origin of the trouble (the inflammation of the tubes being an extension of the inflammation from the endometrium), the galvano-puncture aims at a cure by bringing the current into direct contact with the diseased structures. It may not be essential to actually penetrate to the interior of the tube, unless it is distended, and then, where feasible, it is better to draw off the contents with a fine canula and introduce the current through the canula afterwards for its effects upon the tube. In many instances an inflamed tube may be cured by the intra-uterine galvanization alone, especially if the applications are localized in the cornua of the uterine cavity on that side, and the applications are persisted in. It is sometimes possible even to employ a distended tube in this way where the proximal end is only obstructed by an inflammatory condition. By relieving this condition, drainage into the uterine cavity can be accomplished. But if the tube is flexed at this point and dragged down by its increased weight, or fixed in a prolapsed position by an inflammatory deposit or adhesions, it will be impossible to effect drainage in this way until the distended tube has been emptied and the inflammatory condition and adhesions have been removed. Frequently when the prolapsed and distended tube has been emptied by the method which I have designated, galvano-tapping, the tube is relieved of the weight which drags it down, and, if there be no adhesions, it will rise again to somewhat its normel position and the intra-uterine applications will enable any subsequent drainage to take place by the natural channel into the uterine cavity.

But the condition will not be appropriate for aspiration and drainage by the vagina unless the tube is greatly distended and close to the vaginal wall within easy reach; for I limit the penetration of these aspirations to one centimetre in depth, and deem a puncture which does not exceed this to be perfectly safe if the location has been properly selected. It is seldom necessary to exceed a depth of half a centimetre, but the sheath on the canula is arranged so as to allow a penetration of one centimetre when necessary.

Observing the same antiseptic precautions as for puncture, the point of the trocar is drawn within the canula and passed along the finger in the vagina which guides it to the spot selected. The needle must penetrate easily so as not to endanger rupture of the tube, and, if it does not, the handle of the trocar may be connected with the negative pole and 15 or 20 M. turned on to facilitate its entrance. Withdrawing the trocar when the canula has penetrated to the distance allowed by the sheath, the sac is emptied with the aid of the aspirator.

If the case be one of hydrosalpinx, the negative pole is then connected with the canula, and 50 M. is used for five minutes. If it be pyosalpinx, the cavity is washed out with an antiseptic solution, as an extra precaution, and the positive pole is used through the canula with 50 M. for five minutes. The effect of the current is to cauterize the track of the canula, shutting it off from the tissues which are penetrated, thereby preventing extravasation and the absorption of septic material. At the same time an exit is furnished for subsequent drainage until the puncture closes.

I have been able by this means to relieve and cure a number of cases where otherwise a removal of the diseased structures would have been necessary, and that, too, without risk to the patient and without a prolonged confinement in bed.

The vaginal galvano-puncture is particularly applicable to those cases which are designated as fulness and thickening in the broad ligament, the condition being one that was formerly known as chronic pelvic cellulitis, and which really is a mass of exudation with adhesions surrounding the tube and ovary, which are or have been in a state of chronic inflammation. Here the puncture which brings the current directly into contact with the diseased condition will accomplish what will not be possible by any other means. And the reason is obvious, for by it there is obtained the polar, as well as the interpolar, action of the current. By the other methods of application, the interpolar action alone is made use of. In this we have as much *plus* the polar action. By the method which makes use of the interpolar action only, it is possible to cure some of these cases; but it is slow and often ineffectual, and there is more satisfaction in combining the two.

If I have omitted to describe all of the conditions under this head for which this treatment would be appropriate, I trust I may be pardoned, because of the limited time at my disposal.

